# The role of the PI3K-Akt signaling pathway in the developmental competence of bovine oocytes

**DOI:** 10.1371/journal.pone.0185045

**Published:** 2017-09-18

**Authors:** Gabriella Mamede Andrade, Juliano Coelho da Silveira, Claudia Perrini, Maite Del Collado, Samuel Gebremedhn, Dawit Tesfaye, Flávio Vieira Meirelles, Felipe Perecin

**Affiliations:** 1 Veterinary Medicine Department, Faculty of Animal Sciences and Food Engineering, University of Sao Paulo, Pirassununga, São Paulo, Brazil; 2 Large Animal Hospital, Reproduction Unit, Università degli Studi di Milano, Lodi, Italy; 3 Institute for Animal Sciences (ITW), University of Bonn, Bonn, Germany; China Agricultural University, CHINA

## Abstract

The ovarian follicle encloses oocytes in a microenvironment throughout their growth and acquisition of competence. Evidence suggests a dynamic interplay among follicular cells and oocytes, since they are constantly exchanging “messages”. We dissected bovine ovarian follicles and recovered follicular cells (FCs—granulosa and cumulus cells) and cumulus-oocyte complexes (COCs) to investigate whether the PI3K-Akt signaling pathway impacted oocyte quality. Following follicle rupture, COCs were individually selected for *in vitro* cultures to track the follicular cells based on oocyte competence to reach the blastocyst stage after parthenogenetic activation. Levels of PI3K-Akt signaling pathway components in FCs correlated with oocyte competence. This pathway is upregulated in FCs from follicles with high-quality oocytes that are able to reach the blastocyst stage, as indicated by decreased levels of *PTEN* and increased levels of the *PTEN* regulators bta-miR-494 and bta-miR-20a. Using PI3K-Akt responsive genes, we showed decreased *FOXO3a* levels and *BAX* levels in lower quality groups, indicating changes in cell cycle progression, oxidative response and apoptosis. Based on these results, the measurement of levels of PI3K-Akt pathway components in FCs from ovarian follicles carrying oocytes with distinct developmental competences is a useful tool to identify putative molecular pathways involved in the acquisition of oocyte competence.

## Introduction

The success of *in vitro* production of bovine embryos is closely associated with oocyte quality [1, 2]. The oocyte’s competence to reach the blastocyst stage is progressively acquired during follicle growth [3], and is the result of interactions between external factors and the follicular microenvironment [4]. The ovarian follicle is composed of three cell types in addition to the oocyte, theca, granulosa, and cumulus cells, along with a basement membrane and follicular fluid. Each one of these cell types plays an active role in oocyte differentiation and regulation [5]. Due to its complexity, a reliable method for measuring oocyte competence prior to phenotype analysis following fertilization is not available.

Several morphological, ultrastructural, and metabolic criteria have been used to predict oocyte competence. These morphological criteria remain subjective and, in some cases, are invasive and/or poorly correlated with oocyte competence. Identification of other relevant biomarkers to accurately predict oocyte quality is the primary goal [6]. The appropriate interplay between oocyte and follicular cells (FCs) is indispensable for proper folliculogenesis, oocyte development, and progression to ovulation, as well as subsequent embryo development [7]. Hence, an understanding of the different signaling pathways in FCs that modulate oocyte competence is important for unravelling the key intracellular events driving oocyte competence acquisition.

The phosphatidylinositol-3-kinase/protein kinase B (PI3K-Akt) pathway plays a key role in cellular responses to cell proliferation, apoptosis, DNA repair, and protein synthesis. Based on accumulating evidence, PI3K-Akt signaling is associated with ovarian function, including the recruitment of primordial follicles, granulosa proliferation, corpus luteum survival, and oocyte maturation [8–10]. PI3K-Akt regulators have several functions, including the upregulation of Akt activity by its phosphorylation as a result of PI3K upregulation and downregulation by PTEN (phosphatase homologous to tensin). Additionally, miRNAs are also involved in the regulation of PI3K-Akt signaling, representing an additional layer of complexity in the system regulating this pathway [11]. Specifically, some miRNAs, such as miR-494 and miR-20a, have been shown to exert modulatory effects on *PTEN* levels and, thus, on genes downstream of the PI3K-Akt pathway [12, 13].

Here, we investigated the hypothesis that oocyte competence is associated with alterations in levels of PI3K-Akt pathway components and their regulatory miRNAs during follicle development. Therefore, we dissected ovarian follicles and retrieved FCs and cumulus-oocyte complexes (COCs). We placed the COCs in individual droplets and tracked maturation and parthenogenetic development until the blastocyst stage was reached. Then, we further determined the levels of PI3K-Akt components in previously collected FCs from the original follicles and correlated their levels with the oocyte’s competence to mature and support early development. We determined the levels of genes downstream of PI3K-Akt signaling that are related to different cellular processes (i.e., protein synthesis, DNA repair, cell cycle progression, and apoptosis), as well as the levels of the *PTEN* regulators bta-miR-494 and bta-miR-20a. We also validated the interaction between bta-miR-494 and the bovine *PTEN* 3’UTR (untranslated region) using a luciferase assay. Based on our results, the PI3K-Akt pathway is differentially regulated in FCs, and, as a result, potentially drives oocyte competence.

## Materials and methods

This study was approved by the University of São Paulo Research Ethics Committee (Protocol number 14.1.674.74.0). Experiments were conducted in accordance with International Guiding Principles for Biomedical Research Involving Animals (Society for the Study of Reproduction). Unless indicated otherwise, the reagents and culture media used in this study were purchased from Sigma-Aldrich Chemical Company (St. Louis, MO, USA).

We developed an experimental model based on the individual maturation of oocytes and culture of embryos to determine the levels of PI3K-Akt pathway components in FCs from follicles containing oocytes with distinct development capacities. After maturation and embryo development, FCs were grouped according to oocyte competence. Prior to oocyte maturation, ovarian follicles were dissected, FCs (regardless of cell types) were obtained after centrifugation of the follicular fluid and stored for further use, and COCs were collected for individual IVM and embryo culture after parthenogenetic activation. At the end of the IVM, oocytes were denuded and classified depending on the presence or absence of first polar body in mature (IVM+) or non-mature (IVM-) oocytes, respectively. Regardless of the maturation status, individual denuded oocytes were activated parthenogenetically and placed in a droplet for embryo development *in vitro*. Embryos (zygotes) from the IVM+ group without signs of embryonic cleavage were classified as the ‘non-cleaved’ (NC group). Embryos that started to develop (cleaved) but stopped before reaching the blastocyst stage were classified as the ‘cleaved non-blastocyst’ (CNB group), whereas embryos that had reached the blastocyst stage comprised the ‘blastocyst’ group (BL group). FCs from NC, CNB, and BL groups were used to determine the levels of PI3K-Akt pathway components in samples from follicles containing oocytes with different developmental capacities.

### Oocyte maturation, parthenogenetic activation, and single embryo culture

Ovaries were recovered from slaughterhouses and maintained in a pre-warmed saline solution until follicular dissection. Follicles ranging between 3 to 6 mm diameter in size were dissected. After follicle rupture, COCs were collected for single embryo culture, as described above, and FCs were collected, washed by centrifugation (600 x *g*) to remove the follicular fluid, and immediately placed in liquid nitrogen for future analyses of the PI3K-Akt pathway components to retrospectively determine the follicle of origin. The COCs recovered after follicle dissection were washed with 25 mM HEPES-buffered tissue culture medium-199 (TCM-199; GIBCO, Gaithersburg, MD, EUA) supplemented with 10% (v/v) FCS, 0.2 mM sodium pyruvate, and 50 μg/mL gentamicin.

COCs were matured in individual 9 μL drops of maturation medium (TCM-199) buffered with bicarbonate and supplemented with 10% FCS, 0.2 mM sodium pyruvate, 50 μg/mL follicle stimulating hormone (FSH; Folltropin-V, Bioniche Animal Health Belleville, Canada), and 50 μg/mL human chorionic gonadotropin (hCG) (Vetcore^®^, Hertape Calier), under mineral oil in a 38.5°C incubator with 5% CO_2_ in air.

After 19 hours of IVM, oocytes were individually denuded in 0.2% hyaluronidase in Ca^2+^- and Mg^2+^- free PBS and classified as IVM+ or IVM-, as described above. Cumulus-free oocytes were washed with maturation medium and returned to the incubator for a total of 26 hours of incubation.

All oocytes were activated, regardless of their IVM status. Oocytes were washed thrice with H199 medium (TCM-199 with 25 mM HEPES) containing 0.5 mg/mL bovine serum albumin (BSA), 0.2 mM pyruvate, and 50 μg/mL gentamicin. After washing, oocytes were activated using the same medium supplemented with 5 mM ionomycin for 5 min. After activation, the oocytes were washed thrice with H199 saturated media (TCM-199 with 25 mM Hepes containing 30 mg/mL BSA, 0.2 mM pyruvate, and 10 μg/mL gentamicin) to remove the ionomycin and then washed thrice with synthetic oviduct fluid (SOF) supplemented with 5 mg/mL BSA, 2.5% FCS, 0.2 mM pyruvate, 10 mg/mL gentamicin, and 2 mM 6-dimethylaminopurine (6-DMAP). Finally, the oocytes were placed in 9 μL single droplets of DMAP-supplemented SOF and incubated at 38.5°C with 5% CO_2_ in air for 3 h.

Following activation, oocytes were washed three times with SOF medium supplemented with 5 mg/mL BSA, 2.5% FCS, 0.2 mM pyruvate, and 10 μg/mL gentamicin, and subsequently co-cultured with a monolayer of cumulus cells on the same plate in 9 μL droplets of fresh culture medium covered with mineral oil at 38.5°C in a humid atmosphere of 5% O_2_, 5% CO_2_, and 90% N_2_ for 8 days. Embryos were evaluated at D2 (48 h), D7 (168 h), and D8 (192 h) post-activation. This procedure allowed us to classify oocytes and their respective FCs into three groups according to their development competence: the NC group (mature (IVM+) oocytes that were not cleaved after activation), the CNB group (oocytes that were cleaved but subsequently stopped developing), and the BL group (oocytes that completed their development until they reached the blastocyst stage).

### RNA and protein extraction

Total RNA, including small RNA molecules, and proteins were extracted using either TRI-reagent or TRI-reagent BD (Molecular Research Center, Inc.), according to the manufacturer’s instructions, with some modifications. RNA pellets were dissolved in 10 μL of RNase-free water. Total RNA was treated with DNase I (Life Technologies) to avoid DNA contamination. RNA concentrations were measured using a NanoDrop 2000 (Thermo Scientific) at 260 nm, according to the manufacturer’s protocol. Proteins were precipitated from the organic phase remaining after lysis with TRI reagent and dissolved in 20 μL of an 8 M urea solution before quantification with a Bradford assay (Bio-Rad) according to manufacturer’s protocol.

### Determination of mRNA and miRNA expression using RT-PCR

Briefly, the reverse transcription reaction was performed with 30 ng of total RNA per gene using a High Capacity cDNA reverse transcription kit (Life Technologies), according to the manufacturer’s instructions. PCR amplification was performed with the resulting cDNA using sequence-specific forward and reverse oligonucleotide primers in custom-prepared plates for the indicated genes (Table 1).

**Table 1:** Bovine primer sequences used in the RT-PCR amplification.

Gene symbol	Primer sequences (5’–3’)	Product size	Accession no.	Reference
*ACTB*	FP: CAGCAGATGTGGATCAGCAAGC	91	NM_173979.3	[14]
	RP: AACGCAGCTAACAGTCCGCC			
*BAX*	FP: CCCGAGTTGATCAGGACCAT	153	NM_173894.1	Designed by authors
	RP: CACTCCAGCCACAAAGATGG			
*BCL2*	FP: CTTTGTGGAGCTGTATGGC	119	NM_001166486.1	Designed by authors
	RP: CCAGATAGGCACCCAGGG			
*BRCA*	FP: CTGCATGCCCTGACAGTCCT	132	NM_178573.1	Designed by authors
	RP: CCCCATCGCACGAGTCATCA			
*CDK6*	FP: CTCCGAGGCCTGGACTTTCT	120	NM_001192301.1	Designed by authors
	RP: GATGCGAGCAAGGCCGAA			
*EIF4b*	FP: ACGACTCCAGATCTGCACCTG	103	NM_001035028.2	Designed by authors
	RP: TCTTCACCGTCAATGGCGAGA			
*EIF4e*	FP: TTAATGCCTGGCTGTGACTAC	132	NM_174310.3	Designed by authors
	RP: ACGATCGAGGTCACTTCGTCT			
*FOXO3a*	FP: CGAAGTGGAGCTAGACCCGG	134	NM_001206083.1	Designed by authors
	RP: CGGGGATCATGGAGTCAGCA			
*PI3K*	FP: ACACAGCTGACGGGACCTTT	127	NM_174575.1	Designed by authors
	RP: CCATATTTCCCATCTCGGTGA			
*PTEN*	FP: GCCACAAAGTGCCTCGTTTACC	120	XM_613125.6	[15]
	RP: AGAAGGCAACTCTGCCAAACAC			
*YWHAZ*	FP: GCATCCCACAGACTATTTCC	120	GU817014.1	Designed by authors
	RP: GCAAAGACAATGACAGACCA			

*ACTB*–Beta actin; *BAX*–BCL2-associated X protein; *BCL2* –B cell leucemia/lymphoma 2; *BRCA*–Breast câncer 2; *CDK6* –Cyclin-dependent kinase 6; *EIF4b* –Eukaryotic translation initiation fator 4B; *EIF4e* –Eukaryotic translation initiation fator 4E; *FOXO3a* –Forkhead box O3; *PI3K* –Phosphatidylinositol 3-kinase; *PTEN*–Phosphatase and tensin homolog; *YWHAZ*–Tryptophan 5-monooxygenase activation protein, zeta

Relative quantification of transcripts was performed by qPCR using ABI-7500 (Applied Biosystems) with a denaturation step at 95°C for 10 min, followed by 45 cycles of 95°C for 15 s, 60°C for 1 min, and 95°C for 15 s. A melting curve analysis was performed to ensure the specificity of the products generated. Raw Ct values were normalized to *ACTB*, as previously described for similar tissues [14], to identify the differences among competence groups. Normalized results of quadruplicate samples were used to calculate the relative expression using 2^-ΔCt^ transformation.

For miRNA-494 and miR-20a, the reverse transcription reaction was performed with 100 ng of total RNA using the miScript PCR Starter Kit (Qiagen, Venlo & Limburg, The Netherlands), according to the manufacturer’s instructions. RT-PCR was performed using QuantiTec SYBR Green PCR Master Mix (Qiagen, Venlo, Limburg, Netherlands), 10 μM universal reverse primer (Qiagen, Venlo, Limburg, the Netherlands), 0.1 μL of cDNA and specific primers: miRNA-494 5’ TGAAACATACACGGGAAACCTC 3’ and miRNA-20a 5’ TAAAGTGCTTATAGTGCAGGTAG 3’. Real-time PCR as performed using ABI-7500 (Applied Biosystems). PCR cycling conditions were 95°C for 5 min and 45 cycles of 10 s at 95°C, 55°C for 15 s, and 72°C for 15 s, followed by a melting curve analysis to confirm the uniqueness of the cDNA amplification products. The Ct values of bta-miR-494 and bta-miR-20a, were normalized to the geometric mean of two small reference RNAs (miR-99b and RNU1) and the relative expression was calculated using the 2^-ΔCt^ transformation. The assays were analyzed in quadruplicate and only Ct values less than 35 were considered.

### Target gene validation using a luciferase reporter assay

We constructed a plasmid vector with a fragment of the 3’- UTR harboring the conserved binding sites for bta-miR-494 (wild type construct) or a fragment with mutant variants of the miRNA binding sequences (Mutant construct) inserted between the *SacI* and *XhoI* restriction sites downstream of pmirGLO Dual-Luciferase miRNA Target Expression Vector (Promega, Madison, USA) to experimentally validate the miRNA-mRNA interaction between bta-miR-494 and the *PTEN* gene. Plasmids were amplified and sequenced to verify the presence and absence of miRNA binding sites in the wild type and mutant constructs, respectively. Ovaries were obtained from local slaughterhouses, and granulosa cells were aspirated from small growing follicles and cultured in 24-well plates as previously described [16]. Sub-confluent cells were co-transfected with either the wild type construct or the mutant construct in the presence or absence of a bta-miR-494 mimic using the Lipofectamine 2000 transfection reagent (Invitrogen, Darmstadt, Germany). After 24 h, treated cells were lysed by adding 75 μL of 1X passive lysis buffer (PLB) and shaking for 15 min at room temperature. The activities of firefly luciferase and Renilla luciferase were determined using a Dual-Glo luciferase assay kit (Promega, Madison, WI, USA) by dispensing 20 μL of the protein lysate followed by 100 μL of luciferase assay reagent (LAR) and 100 μL of Stop & Glo reagent. Data were calculated as the ratio of firefly luciferase activity to Renilla luciferase activity and compared with the group transfected with empty vector alone.

### Akt protein detection and quantification by western blotting

Samples were separated on 12% SDS-polyacrylamide gels to detect phosphorylated-Akt (p-Akt) and β-Actin. Proteins were then transferred to a PVDF membrane using Bio-Rad products and system (Trans-Blot^®^ Turbo-Bio-Rad). These membranes were blocked with 5% BSA (Sigma-Aldrich) in TBST (100 mM NaCl, 0.1% Tween 20, and 50 mm Tris, pH 7.4) for 1 h and incubated with the primary antibody in TBST containing 1% BSA overnight at 4°C. The primary antibody was a p-Akt1/2/3 antibody (Ser 473) (a rabbit polyclonal IgG from Santa Cruz Biotechnology, 1:200 dilution, catalogue number SC-101629), which detects a ~60 kDa protein. Blots were then washed three times with TBST, incubated with an HRP-labelled anti-rabbit IgG (catalogue number #7074S - Cell Signaling Technology) in TBST containing 1% BSA for 1 h at room temperature, and washed another three times with TBST. Finally, blots were washed three times with TBST and incubated with an HRP-labelled mouse monoclonal anti-beta-actin (ACTB) antibody (catalogue number A3854—Sigma-Aldrich) that specifically detects a 43 kDa proteins for 3 h at room temperature to normalize the data. Blots were washed another three times with TBST and treated with Clarity^™^ Western ECL substrate (Bio-Rad) to visualize the proteins.

### Statistical analyses

Data are displayed as the means ± SEM of replicate samples. Statistical analyses were performed using the SAS 9.3 software (SAS Institute). ANOVA was performed to assess statistically significant differences, respecting normality and homoscedasticity premises. Means were compared using Duncan’s test. When ANOVA premises were not satisfied, data were compared using the Kruskal-Wallis test. Unless stated otherwise, a level of 5% was considered significant.

## Results

### Individual oocyte competence and developmental rates

One hundred seventy-four oocytes were collected and individually activated. Among these oocytes, 63.79% cleaved and 29.31% reached the blastocyst stage (S1 Table). Only routines in which both blastocyst/cleaved rates were greater than 35% were used in the following experiments.

### Analysis of *PTEN* and *PI3K* transcripts and p-Akt protein levels in fcs in relation to oocyte competence

**Fig 1:**
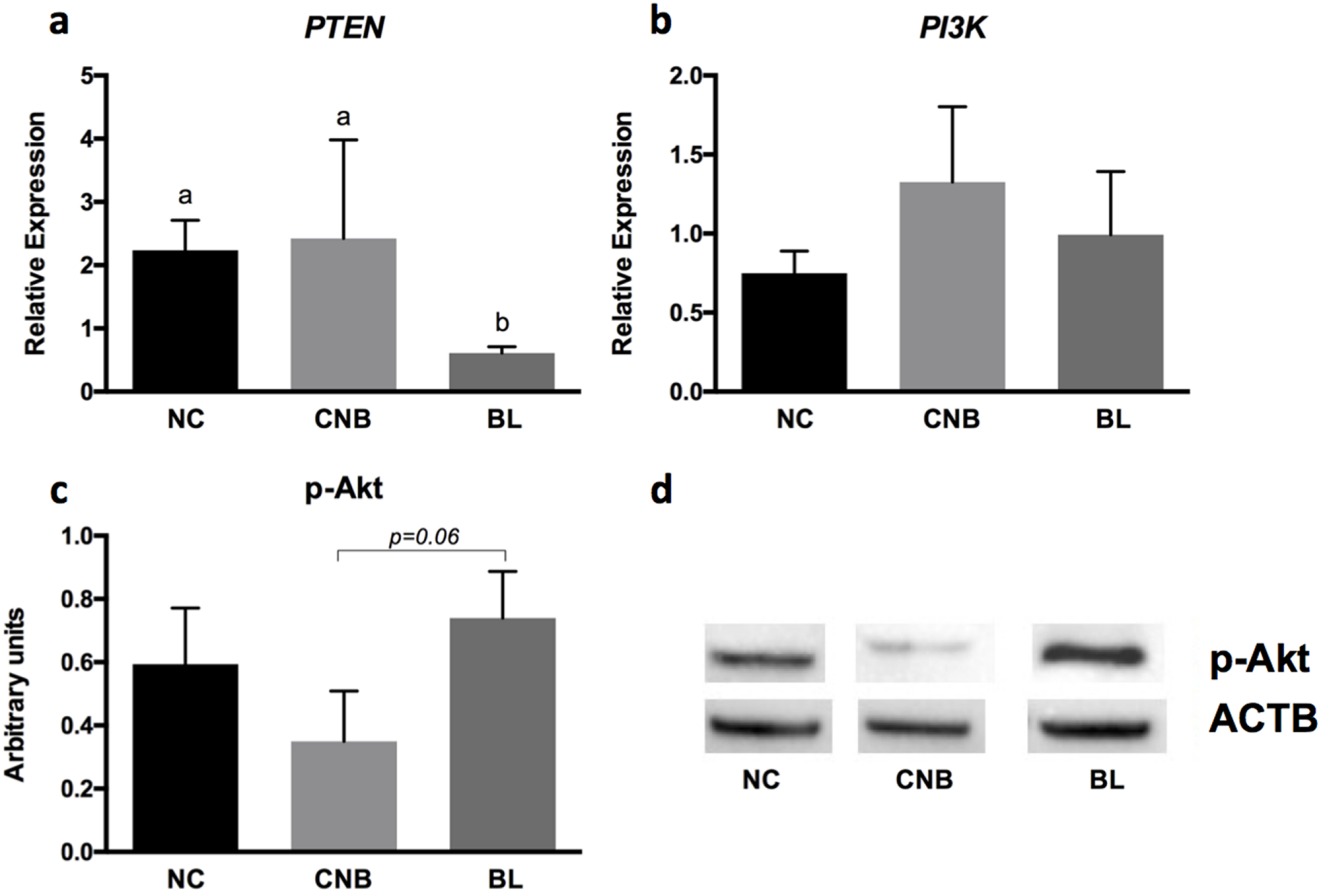
Relative expression of (A) *PTEN* and (B) *PI3K* mRNAs in follicular cells according to oocyte competence. (C) Levels of the phosphorylated Akt protein (p-Akt) obtained from western blotting analyses and (D) representative protein bands for p-Akt (~ 60 kDa) and Actin B (ACTB; 42 kDa) from western blotting analyses. Non-cleaved (NC), cleaved non-blastocyst (CNB), blastocyst (BL). Bars depict means and error bars depict standard errors of the means. Differences were statistically significant when p < 0.05.

Messenger RNA levels of *PTEN* (a negative effector of the PI3K-Akt pathway) and *PI3K* (a positive effector) were determined in FCs from the NC, CNB, and BL groups. The relative abundance of the *PTEN* mRNA was significantly reduced (p < 0.05) in FCs that originated from follicles containing oocytes capable of generating blastocysts *in vitro* compared to FCs that originated from follicles containing oocytes that did not reach this stage (Fig 1A). PTEN is responsible for converting PIP3 to PIP2 and maintaining low levels of PIP3, an Akt activator. Although *PI3K* mRNA levels appeared to increase in CNB and BL samples, no significant differences among the oocyte quality groups were detected (Fig 1B). PI3K is responsible for converting PIP2 to PIP3 and, therefore, is an important activator of this pathway.

The PI3K-Akt signaling pathway is modulated by the levels of PTEN and PI3K, which are responsible for Akt activation. We evaluated the levels of the phosphorylated Akt protein (p-Akt) in FCs within the distinct oocyte quality groups to determine the mechanism by which the PI3K-Akt pathway influences oocyte competence. The western blot analysis revealed increased levels of p-Akt (p < 0.06) in FCs originating from follicles that generated blastocysts compared to FCs that cleave but interrupt their development after the cleavage (Fig 1C).

### *MiR-494* and *miR-20a* analysis in fcs in relation to oocyte competence

**Fig 2:**
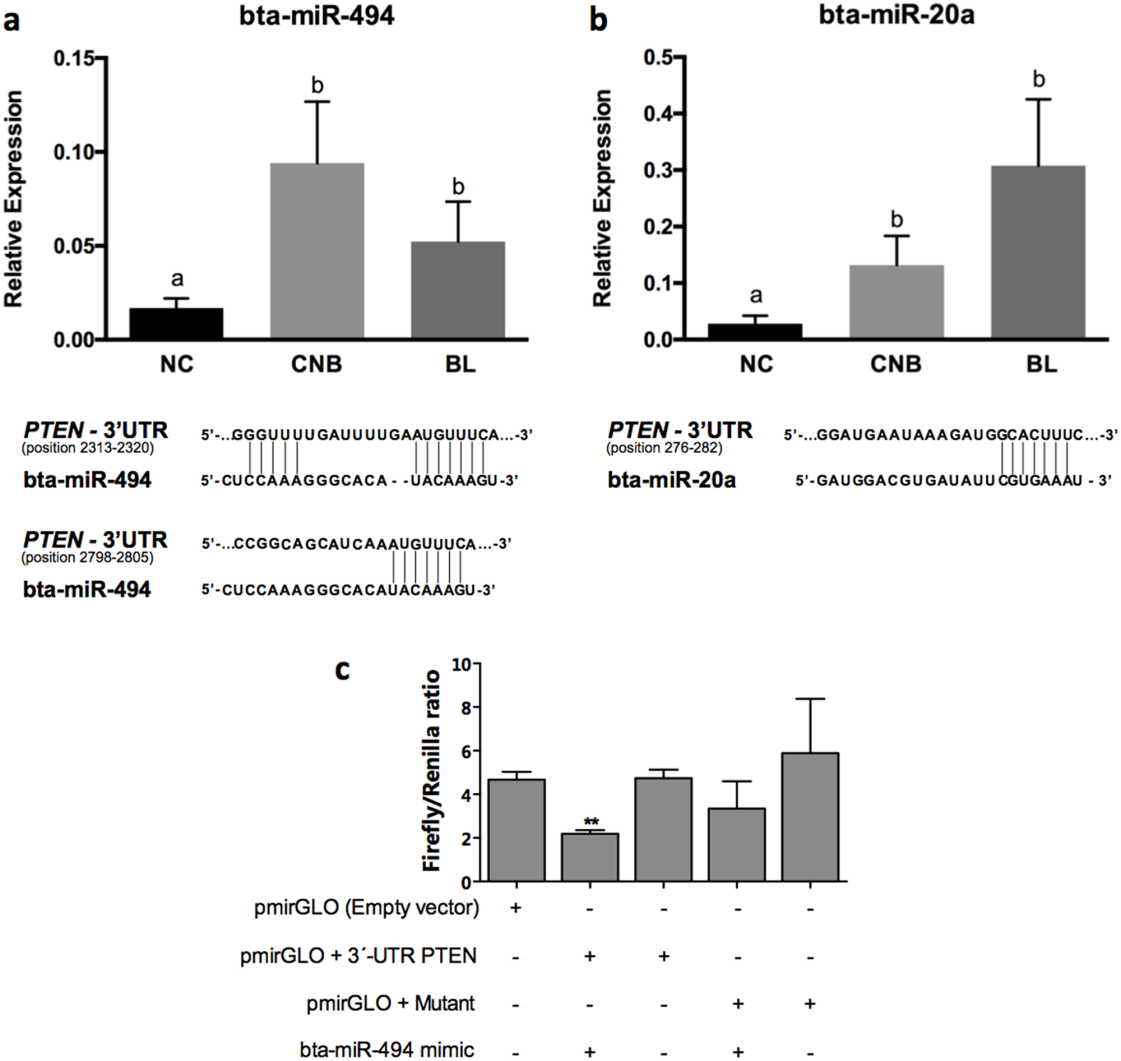
Relative levels of bta-miR-494 and bta-miR-20a in follicular cells. (A) Relative levels of bta-miR-494 in non-cleaved (NC), cleaved non-blastocyst (CNB), and blastocyst (BL) groups. Bovine miRNA-494 and its validated target *PTEN*. A bioinformatics analysis of the 3’UTR of *PTEN* revealed two conserved target sites among mammals. (B) Relative levels of bta-miR-20a in non-cleaved (NC), cleaved non-blastocyst (CNB), and blastocyst (BL) groups. (C) Luciferase assay of the bovine *PTEN* 3’UTR in granulosa cells. Firefly luciferase activity was evaluated in cells transfected the empty pmirGLO vector, the vector with the wild type *PTEN* 3’UTR insert, and the vector with the mutated insert following the transfection of the bta-miR-494 mimic. Different letters indicate p<0.05. ** indicate p<0.01.

We investigated the levels of bta-miR-494 and bta-miR-20a, two validated regulators of *PTEN* in humans and bovine, respectively (Liu et al. 2010; Andreas et al. 2016). *PTEN* contains a validated and conserved binding site for miR-494 in many mammalian species, including bovine, according to TargetScan (http://www.targetscan.org/). Additionally, bta-miR-20a was recently validated as a regulator of *PTEN* in bovine granulosa cells [13]. Relative levels of bta-miR-494 and bta-miR-20a were significantly higher (p < 0.05) in FCs derived from follicles in which the oocytes cleaved, regardless of subsequent blastocyst generation (CNB and BL), than in follicles in which the oocytes did not cleave (NC) (Fig 2A and 2B). Additionally, we performed a luciferase assay to determine whether miR-494 regulated *PTEN* by binding to its 3’UTR. Granulosa cells containing the vector with the wild type copy of the *PTEN* 3’UTR displayed decreased firefly luciferase activity after transfection of the miR-494 mimic compared to the control and mutant construct, suggesting that miR-494 is a validated regulator of *PTEN* (Fig 2C).

### Analysis of PI3K-Akt-regulated genes in FCs according to oocyte competence

The PI3K-Akt signaling pathway regulates different cellular effects, including changes in protein synthesis, DNA repair, cell cycle progression, apoptotic signaling etc. We chose to analyze downstream genes related to different cellular effects using RT-PCR to identify the mechanisms by which PI3K-Akt signaling regulates oocyte competence.

**Fig 3:**
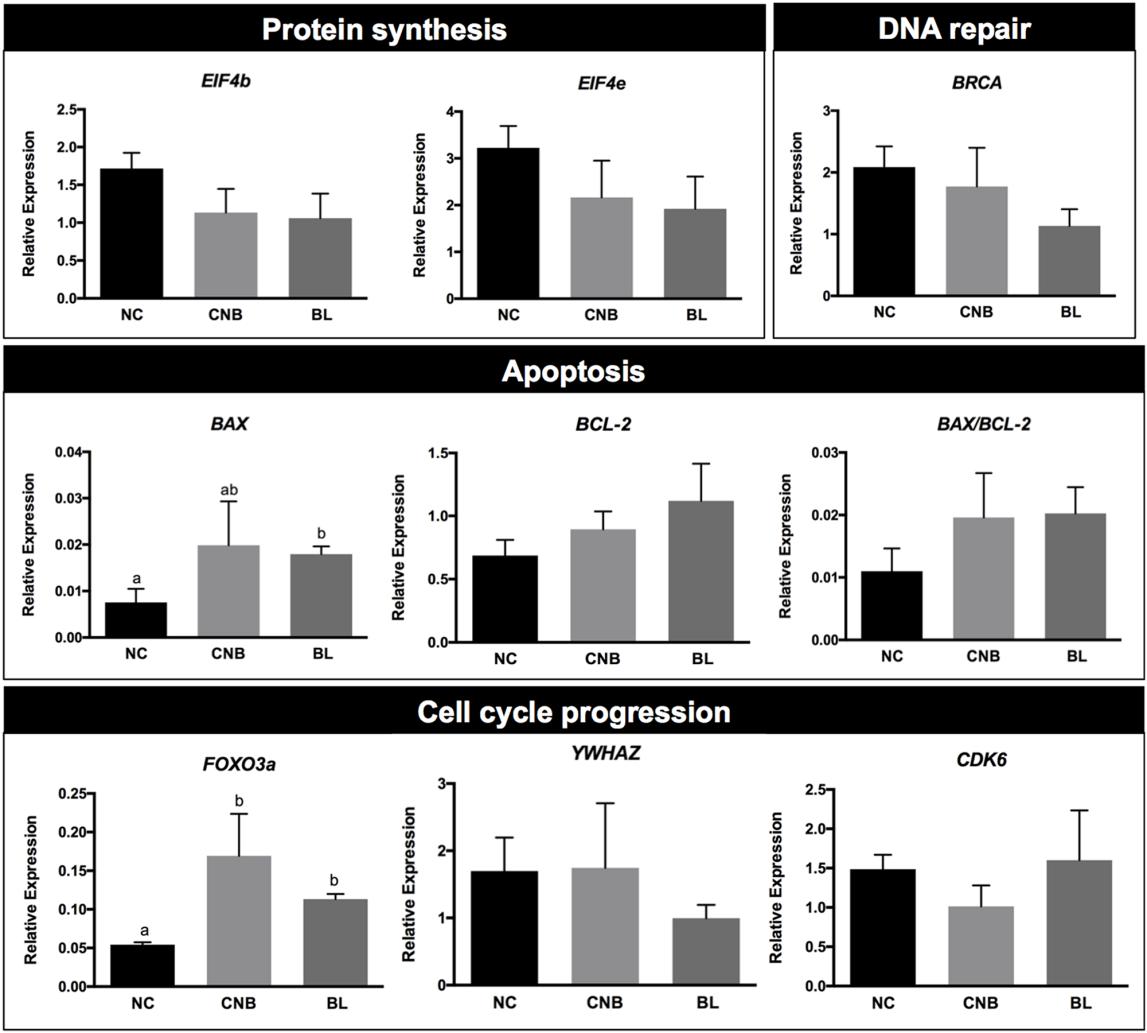
Relative expression of mRNAs for genes responsible for the downstream cellular effects of PI3K-Akt signaling on non-cleaved (NC), cleaved non-blastocyst (CNB) and blastocyst (BL) groups. Bars depict means and error bars depict standard errors of the means. Different superscripts (a and b) indicate significant differences (p < 0.05).

We analyzed the mRNA levels of 8 genes (*BAX*, *BCL2*, *BRCA*, *CDK6*, *EIF4b*, *EIF4e*, *FOXO3a*, and *YWHAZ*) in FCs from the different oocyte competence groups (Fig 3). Among these genes, only the levels of *BAX* and *FOXO3a* showed significant differences between the competence groups. *BAX* levels were increased (p < 0.05) in the BL group, although the ratio between *BAX/BCL-2* transcript levels did not differ among the groups. Similarly, *FOXO3a* mRNA levels were significantly increased (p < 0.05) in the BL and CNB groups compared to the NC group, implying a possible FOXO3a-dependent cell cycle progression response.

## Discussion

We dissected ovarian follicles, stored FCs, and individually activated oocytes to evaluate their developmental competences and determine whether components of the PI3K-Akt pathway serve as predictors of oocyte quality. *In vitro* parthenogenetic development allowed us to pool FCs into different groups according to oocyte competences using a retrospective method for subsequent RNA and protein assays. That the main components of the PI3K-Akt signaling pathway displayed different levels of mRNA or protein expression, depending on oocyte quality. In addition, we investigated genes regulated by PI3K-Akt signaling and observed an increase in *FOXO3a* levels, suggesting the existence of mechanisms governing the modulation of cell-cycle progression, protection against oxidative stress, and the anti-apoptotic response.

Oocyte growth and follicle development rely on the regulation of pathways involved in cell proliferation and survival, such as the PI3K-Akt signaling pathway [17]. The recruitment of primordial follicles is affected by the proliferation of granulosa cells and the regulation of the interaction between the Notch and PI3K-Akt pathways [17, 18]. Follicle loss, maintenance, and growth are strictly controlled by complex molecular interactions, including PI3K-Akt signaling. Stimulation of PI3K promotes Akt phosphorylation, resulting in follicle survival and the induction of growth. Alternatively, this pathway is suppressed by the action of PTEN [18]. PTEN is involved in converting PIP3 to PIP2, and high levels of PTEN are responsible for decreasing the Akt pathway activity, subsequently reducing cell proliferation [17, 19].

In the present study, we detected higher levels of the PTEN mRNA and lower levels of bta-miR-494 and bta-miR-20a, two validated modulators of *PTEN*, in FCs from poor oocyte quality groups, suggesting that the PI3K-Akt signaling pathway is less active in these follicles. The inhibition of PTEN in human ovary *in vitro* results in the increased activation of primordial follicles, but compromises the development of growing follicles [18]. In the murine ovary, the deletion of PTEN in mouse oocytes resulted in pan-ovarian follicle activation and premature oocyte depletion, whereas granulosa cell-specific disruption of PTEN did not affect the initiation of follicle growth, but increased GC proliferation and enhanced ovulation [9, 20]. PTEN was also down-regulated by the up-regulation of miR-494 (a conserved miRNA among mammals) in human bronchial cells, suggesting that this mechanism is important in controlling PTEN levels [21]. Furthermore, we validated the interaction between miR-494 and PTEN using a luciferase assay and the wild-type or mutant 3’UTR. As predicted by the bioinformatics analysis, binding of bta-miR-494 to the wild-type 3’UTR sequence reduced the levels of luciferase activity compared to the control and the PTEN mutant sequence. Therefore, bta-miR-494 regulates the levels of *PTEN* in follicular cells. Similar analysis were performed for bta-miR-20a, which was validated as a regulator of *PTEN* in granulosa cells [13]. Thus, o *PTEN* levels are regulated by the increase in miR-494 and miR-20a expression in follicles harboring competent oocytes. Additionally, multiple miRNAs regulating the same mRNA target are necessary required to induce a physiological response, such as upregulation of Akt, which was observed in cells obtained from follicles associated with higher quality oocytes.

Follicular cells from follicles harboring incompetent oocytes have a molecular profile marked by an increase in *PTEN* expression and lower expression of bta-miR-494 and bta-miR20a, which indicates lower activity of the PI3K-Akt pathway. In contrast, the competent group display different levels of these indicators, confirming the increased activity of the PI3K-Akt pathway in this group. These two molecular profiles are associated with oocyte competence, and based on previously described functions of the PI3K-Akt pathway in follicular environments, we postulate that these differences in oocyte competence are related to the regulation of cell proliferation, protection from oxidative stress, and inhibition of apoptosis in the FCs, as discussed below.

In addition to its involvement in cell proliferation and survival, the PI3K-Akt signaling pathway plays roles in apoptosis, protein synthesis, the cell cycle, angiogenesis, DNA repair, and metabolic pathways, as reviewed in a previous publication [22]. In the present study, we analyzed the mRNA levels of genes related to different cellular processes, such as *BAX*, *BCL-2* (apoptosis), *BRCA* (DNA repair), *EIF4b*, *EIF4e* (protein synthesis), *FOXO3a*, *YWHAZ* (cell cycle progression), and *CDK6* (FOXO-independent cell cycle progression) to identify the intracellular events that mediate the relationship between the PI3K-Akt signaling pathway and the ability of the oocyte to develop into a blastocyst. We observed differences in the levels of the *FOXO3a* and *BAX* transcripts between FC groups, which were associated with different oocyte capacities to develop into blastocysts.

Here, *FOXO3a* levels were increased in FCs of higher quality oocytes. The transcription factor FOXO3a plays an important role in ovarian activity [23], and, when activated, it has broad anti-proliferative and pro-apoptotic cellular effects. In addition to these effects, FOXO3a protects quiescent cells from oxidative stress [24]. Moreover, FOXO3a has also been reported to protect mouse oocytes from oxidative stress [25]. The effects induced by increased FOXO3a expression may also vary, depending on the signal integration and associated factors, and may range from triggering cell death to promoting survival by inducing the expression of oxidative stress resistance factors [26]. Since this study does not include assessments of other factors involved in regulating FOXO3a levels, with the exception of the p-Akt protein and *FOXO3a* levels, we cannot specifically identify the final effects associated with FOXO3a in FCs. However, since different levels of *FOXO3a* were detected in FCs from different oocyte competence groups, we propose that the Akt-FOXO3a interaction functions to adjust the regulation of the oxidative stress response and cell proliferation and leads to apoptosis by either favoring or preventing the acquisition of competence. We also hypothesize that in the BL group, the Akt-FOXO3a interaction promotes the transcription of *FOXO3a* (since it acts as an enhancer for its own transcription), which then contributes to the regulation of ROS scavengers. Moreover, Akt, which inhibits FOXO3a, allows a final proliferative and anti-apoptotic response in high competence follicles.

Another gene that displayed differences in expression levels among the developmental competence groups was *BAX*, which was increased in the higher quality group. *BCL2* and *BAX* are anti-apoptotic and pro-apoptotic genes, respectively, and serve as markers of apoptosis in ovarian tissues [27]. Oocyte competence may be related to events linked to apoptosis, and early apoptosis signals in the oocyte [28] or in GCs [29] are positively correlated with oocyte competence, suggesting the involvement of apoptotic processes in follicular and oocyte development. The pro-apoptotic or anti-apoptotic effects of *BCL-2* and *BAX* genes depend on protein-protein interactions. The BAX protein forms homodimers or heterodimers with BCL-2. The increase in the proportion of BAX homodimers in relation to BAX-BCL-2 heterodimers determines the triggering of cell death through apoptosis. Thus, the *BAX/BCL-2* ratio is the main indicator of the apoptotic process [30, 31]. In this study, the *BAX/BCL-2* ratios were not significantly different among oocyte quality groups, and therefore, we do not have clear evidence that this ratio regulates the acquisition of oocyte competence, as previously suggested by Yang and collaborators [32]. The homodimerization of BAX and its translocation to the mitochondria to trigger apoptosis is directly regulated by the p-Akt protein [33]. The p-Akt protein phosphorylates the BAX protein and regulates its activity; thus, BAX phosphorylation prevents its translocation to the mitochondria and induces its maintenance in the cytoplasm as an heterodimer with BCL-2, preventing apoptosis. In the results described in this study, the BL group, in addition to displaying higher *BAX* levels, also displayed elevated p-Akt level. Therefore, in these cells, the elevation of the *BAX* mRNA did not lead to a higher incidence of apoptosis, due to its low activity resulting from the phosphorylation induced by p-Akt. Furthermore, in the CNB group, BAX expression, but not p-Akt, is elevated in the FCs, potentially suggesting a higher probability of apoptosis, even if the *BAX/BCL-2* ratio does not increase. Finally, p-Akt also helps control apoptosis by regulating BCL-2 availability. BCL-2 and its associated death promoter (BAD) form heterodimers, precluding BCL-2 from forming heterodimers with BAX. Phosphorylation of BAD by p-Akt leads to the isolation of BAD by the 14-3-3 protein family and a subsequent anti-apoptotic effect [34]. Therefore, p-Akt, acting by either phosphorylating and sequestering BAD or by phosphorylating BAX and inducing its localization in the cytoplasm, triggers the anti-apoptotic response within cells.

**Fig 4:**
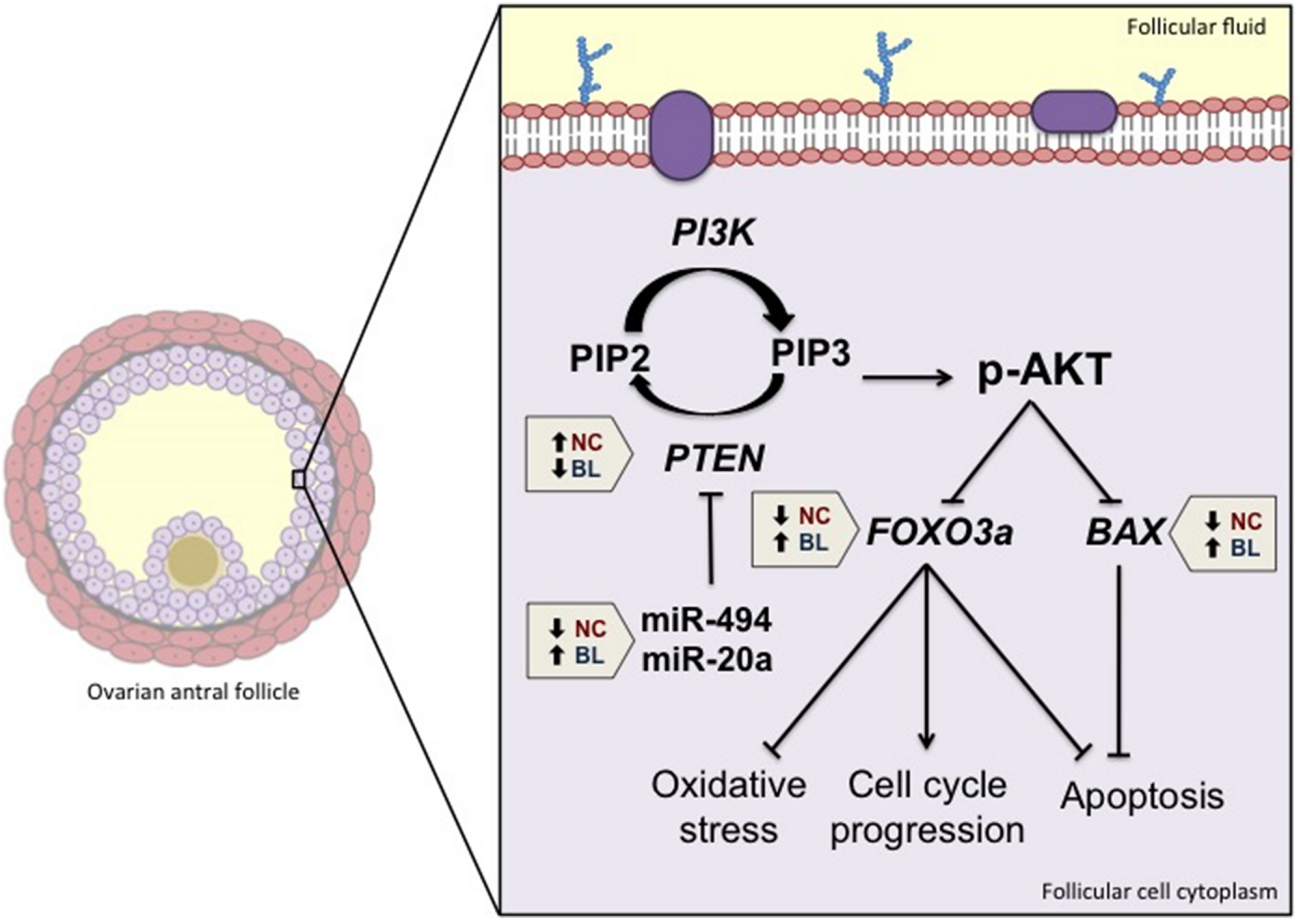
Schematic representation of the PI3K-Akt signaling pathway model and cellular responses in ovarian follicular cells (FCs) associated with competent oocytes that were able to reach the blastocyst stage (BL) and incompetent oocytes that were unable to initiate embryo development after maturation (NC). Pentagons indicate whether a given component of the pathway was upregulated (upward arrow) or downregulated (downward arrow) in FCs from the BL and NC groups. Lines ending in arrowheads indicate stimulation, whereas lines ending in bars indicate blockage.

The mechanisms involved in the acquisition of oocyte competence have not yet been completely elucidated. The acquisition of oocyte competence is a multifactorial process, and although some basic processes are well known, such as the progression of meiosis and migration of cortical granules, some intracellular processes and molecular events that lead to the formation of an oocyte capable of supporting development are still unclear. Several factors influence oocyte quality and regulate competence acquisition. However, an adequate follicular microenvironment is a key factor in successful follicle growth and oocyte competence acquisition. In conclusion, using a retrospective developmental competence model, we investigated whether the PI3K-Akt pathway was associated with oocyte quality. This model allowed us to compare the molecular profiles of components of the PI3K-Akt signaling pathway in FCs with oocyte competence levels ranging from development to the blastocyst stage. We were able to identify a molecular landscape formed by the PI3K-Akt pathway in FCs consisting of lower *PTEN*, *FOXO3a*, bta-mir-494, bta-miR-20a, and *BAX* levels, leading to high oocyte developmental potential (Fig 4). This molecular landscape is compatible with cellular responses related to protection against oxidative stress, cell proliferation, and inhibition of apoptosis, suggesting that these PI3K-Akt-induced cellular responses contribute to oocyte competence acquisition during oogenesis.

## Supporting information

S1 Table Routines for individual parthenogenetic bovine embryo development displaying blastocyst/cleaved rates greater than 35%. (DOCX)
