# Supplementary material for: The role of the PI3K-Akt signaling pathway in the developmental competence of bovine oocytes
Source: PLoS One. 2017 Sep 18;12(9):e0185045. doi: 10.1371/journal.pone.0185045 (PMC5602670; doi:10.1371/journal.pone.0185045)
Supplement: S1 Table — (DOCX) [file pone.0185045.s001.docx]

S1 Table. Routines for individual parthenogenetic bovine embryo development displaying blastocyst/cleaved rates greater than 35%.

| **Routine** | **# oocytes** | **# cleaved** | **cleaved** (%) | **# blastocysts** | **blastocysts** (% oocytes) | **blastocysts** (% cleaved) |
| --- | --- | --- | --- | --- | --- | --- |
| 1 | 16 | 10 | 62.50 | 6 | 37.50 | 60.00 |
| 2 | 16 | 9 | 56.25 | 5 | 31.25 | 55.56 |
| 4 | 19 | 11 | 57.89 | 5 | 26.32 | 45.45 |
| 6 | 21 | 15 | 71.43 | 7 | 33.33 | 46.67 |
| 7 | 18 | 11 | 61.11 | 4 | 22.22 | 36.36 |
| 8 | 22 | 16 | 72.73 | 8 | 36.36 | 50.00 |
| 9 | 23 | 17 | 73.91 | 7 | 30.43 | 41.18 |
| 10 | 17 | 11 | 64.71 | 5 | 29.41 | 45.45 |
| 11 | 22 | 11 | 50.00 | 4 | 18.18 | 36.36 |
| **TOTAL** | 174 | 111 | 63.79 | 51 | 29.31 | 45.95 |
